# Robotic Lobectomy Learning Curve Has Better Clinical Outcomes than Videothoracoscopic Lobectomy

**DOI:** 10.3390/jcm13061653

**Published:** 2024-03-14

**Authors:** Pablo Luis Paglialunga, Laureano Molins, Rudith Guzmán, Angela Guirao, Irene Bello, Anna Ureña, Leandro Grando, Nestor Quiroga, Xavier Michavila, Marc Boada

**Affiliations:** 1 Department of Thoracic Surgery, Institut Clínic Respiratori (ICR), Hospital Clinic of Barcelona, University of Barcelona, 08036 Barcelona, Spain; lmolins@clinic.cat (L.M.); rlguzman@tauli.cat (R.G.); guirao@clinic.cat (A.G.); ibello@clinic.cat (I.B.); aurenal@clinic.cat (A.U.); grando@clinic.cat (L.G.); quiroga@clinic.cat (N.Q.); mboada@clinic.cat (M.B.); 2 Institut d’Investigacions Biomèdiques August Pi I Sunyer (IDIBAPS), 08036 Barcelona, Spain

**Keywords:** robotic-assisted thoracic surgery (RATS), video assisted thoracic surgery (VATS), minimally invasive thoracic surgery (MITS), learning curve, lobectomy, lung cancer, surgical techniques

## Abstract

**Introduction**: The robotic-assisted (RATS) lobectomy learning curve is usually measured compared to an established videothoracoscopic (VATS) surgery program. The objective of our study is to compare the learning curves of both techniques. **Methods**: We performed an intention-to-treat analysis comparing the RATS vs. VATS lobectomies. Surgical time, conversions, complications, number of lymph nodes (LNs) and lymph node stations harvested, chest drainage duration, length of stay, readmissions, and 90-day mortality were compared between both groups. The learning curve was assessed using the CUSUM method. **Results**: RATS cases (30) and VATS cases (35) displayed no significant differences. The RATS learning curve was completed after 23 procedures while the VATS curve required 28 interventions. Complications appeared in four RATS procedures and in eight VATS patients. No differences in the number of LNs and harvested LN stations were reported. Four patients were readmitted in the RATS group, and eight in the VATS group. No 90-day postoperative mortality was observed in either group. The RATS group reported fewer chest tube days (3 (2–5) vs. 5 (4–5.8), *p* = 0.005) and hospital days (4 (3–6) vs. 5 (4–6), *p* = 0.023). **Conclusions**: The RATS curve appears shorter than the VATS curve. RATS lobectomies resulted in reduced chest tube duration and length of stay during the learning time period.

## 1. Introduction

Thoracic surgery has undergone significant advances in recent years with the emergence of new minimally invasive surgical techniques. Since the first reported cases of videothoracoscopic surgery in the 1990s [1], VATS has become the preferred approach for early-stage non-small cell lung cancer [2,3]. More recently, the VIOLET randomized controlled trial demonstrated the superiority of VATS over thoracotomy [4]. Taking another step forward, in 2002, Melfi et al. reported the first series of anatomical lung resections using a robotic platform [5] establishing its safety and efficacy.

It is important to emphasize that when comparing both techniques, the robotic approach features greater technical and technological advances, such as wider and more agile instrument movement, three-dimensional visualization of the surgical field, minimal hand tremors, and improved surgeon ergonomics [6,7]. However, these advancements have not yet clearly translated into improvements in clinical outcomes.

In contrast, robotic-assisted thoracic surgery (RATS) has several disadvantages such as the absence of tactile feedback and physical separation to the operative field in the case of bleeding [8]. Consequently, RATS has been regarded as a challenging technique that necessitates longer durations for lung resection surgeries. Moreover, RATS incurs higher economic costs compared to VATS [9]. As a result, certain thoracic surgery departments are hesitant to incorporate RATS into their routine surgical practice.

The learning curve for RATS lobectomy is commonly assessed by comparing it with an established VATS program or by solely considering the variable of the operative time. The aim of our study was to compare the surgical outcomes and learning curves of both techniques, taking into account the multiple factors that affect them.

## 2. Materials and Methods

### 2.1. Study Type

We performed an intention-to-treat analysis comparing the first consecutive robotic-assisted lobectomies performed between January 2021 and March 2022 (RATS group) and the first video-assisted lobectomies performed between October 2016 and April 2018 (VATS group) by the same surgeon using the same surgical approach (tunnel technique).

### 2.2. Demographics and Patients’ Characteristics/Variables Used

Demographics and patients’ characteristics, including age, gender, comorbidities, functional status, lung location, tumor size, induction therapy, centrality, and stage according to the 8th edition of the TNM staging system, were documented prior to surgery. Intraoperatively, the surgical time, conversion rate, and reasons for conversion were recorded. Postoperatively, complications according the Clavien–Dindo classification [10], the number of lymph nodes (LNs) and lymph node stations harvested, and outcomes at discharge, such as readmission within 30 days and 90-day mortality, were also documented. The day of chest tube removal and the day of discharge were registered to calculate chest tube days and the length of hospital stay (LOS).

### 2.3. Selection Criteria

The selection criteria employed during the study did not differ from those utilized for VATS and RATS in our hospital. Comprehensive patient evaluations were conducted by a multidisciplinary thoracic tumor committee. For pulmonary interventions, tumors up to clinical stage IIB were selected for a minimally invasive approach, following the guidelines outlined in the 8th edition of the TNM classification. All patients in both series were 18 years or older, selected for nonextended elective anatomical lung resection (segmentectomy, lobectomy, bilobectomy, or pneumonectomy). In our study, the preoperative physiologic assessment began with a cardiovascular evaluation and spirometry to measure the forced expiratory volume in one second (FEV1) and diffusing capacity for carbon monoxide (DLCO), followed by the calculation of predicted postoperative (PPO) lung functions. Patients with both %PPO FEV1 and %PPO DLCO values above 60% were considered at low risk for anatomic lung resection, without further tests, according to the standardized selection criteria from the American College of Chest Physicians evidence-based clinical practice guidelines [11].

### 2.4. Centrality

Operationally defined here as a tumor growing in the inner third of the thorax on a CT scan [12], tumor centrality is considered an independent risk factor for lymph node metastasis and has been included in the ESTS (European Society of Thoracic Surgeons) guidelines for preoperative lymph node staging for non-small cell lung cancer, as a criterion for invasive preoperative staging with endo bronchial ultra sound (EBUS) and/or mediastinoscopy.

### 2.5. Surgeon’s Expertise and Training in VATS and RATS Anatomical Lung Resections

During the surgeon’s training period, he gained experience in anatomical resections through thoracotomy and VATS under the supervision of senior surgeons. Before starting to use the VATS technique for anatomic lung resection, the surgeon had performed more than 100 low-complexity thoracoscopic procedures. His first resections as a main surgeon are included in this paper.

To begin performing robot-assisted surgeries, the surgeon received Intuitive Surgical certification; his training included online Da Vinci technology modules, simulator exercises (>20 h), and console surgeon training (a 2-day course with hands-on practice). He also received guardianship by a proctor using a second console for the first two cases. Before starting to perform robotic lobectomies, the surgeon performed a few minor RATS resections and more than 100 VATS lobectomies.

### 2.6. Surgical Technique

**Figure 1:**
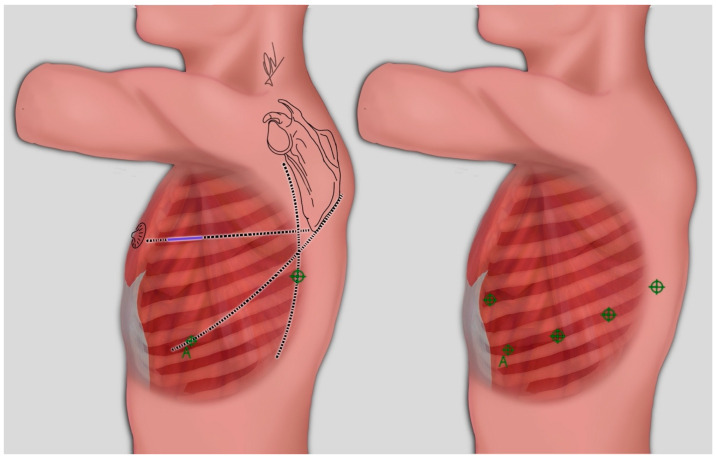
**Left**: Port placement for VATS. 4 cm utility incision (blue line) was made in the 4th intercostal space (ICS). Camera port was placed at the 7th ICS (A), and a 1.5 cm incision was made at the 8th ICS. **Right**: Port placement for RATS lung resection. All ports including assistance (A) were placed in the eighth ICS, except for the anterior port, which was placed in sixth intercostal space. Abbreviations: RATS: robotic-assisted thoracic surgery; VATS: video-assisted thoracic surgery.

The robotic surgeries were performed utilizing the Da Vinci™ Xi surgical system (Intuitive, Sunnyvale, CA, USA/Abex Excelencia Robótica, Madrid, Spain). For all procedures, 30° optics were used. Patients were placed in the lateral decubitus position with lateral flexion of the thorax. The placement of all ports, including the assisting port, was in the eighth intercostal space, except for the anterior port, which was positioned in the sixth intercostal space (Figure 1, right). CO_2_ insufflation was used at low pressure and flow (pressure 5, flow 5) [13].

In the VATS group, we utilized a videothoracoscopy tower along with conventional equipment featuring 30° optics. The patient was placed in the lateral decubitus position, and a 4 cm utility incision was made in the 4th intercostal space (ICS), located in front of the latissimus dorsi muscle, using a soft tissue retractor. Additionally, a low anterior 12 mm camera port was placed at the 7th ICS. Also, a 1.5 cm incision was made at the 8th ICS, in a straight line descending from the scapula and anterior to the latissimus dorsi muscle. It is worth noting that CO_2_ was not utilized in the VATS group (Figure 1, left).

In all cases, all resections were anatomical and met the criteria for a complete resection [14]. Achieving a complete resection necessitates fulfilling several criteria: ensuring clear resection margins confirmed under microscopic examination, performing systematic nodal dissection or lobe-specific systematic nodal dissection, the absence of an extracapsular nodal extension of the tumor, and confirming that the negativity of the highest mediastinal node is removed. In cases where resection margins are involved, extracapsular nodal extension is present, positive lymph nodes remain unresected, or positive pleural or pericardial effusions are detected, the resection is classified as incomplete. At the conclusion of the surgery, a chest tube was inserted through one of the incisions.

### 2.7. Surgical Time

The surgical time was defined as the time from skin opening to skin closure. In the RATS group, this encompassed port placement, robot docking and targeting, console time, specimen retrieval, and finally, skin closure.

### 2.8. Conversion

Conversion occurs when a surgical procedure initiated through RATS is later switched to another approach, such as VATS or open surgery, due to emergent, technical, or oncological reasons. Regardless of the type of conversion, the final surgery and its outcome were duly recorded and included in our comprehensive database. In the VATS group, we considered that a conversion had taken place when the transition was made to an open approach.

### 2.9. Chest Tube and Discharge

In both groups, the same criteria were used to assess pleural drainage removal. In our case, we established a threshold of less than 400 mL of fluid and the absence of an air leak within a 24 h period, with a chest X-ray showing lung expansion. All this was assessed by the surgeon who performed the surgical procedure. Prior to hospital discharge, chest radiography was performed to verify correct lung expansion in all cases.

### 2.10. Statistical Analysis

Statistical analyses were conducted using R version 4.2.2 (R Foundation for Statistical Computing, Vienna, Austria). Categorical data were summarized as frequency counts and percentages. All continuous variables were examined for normality using the Shapiro–Wilk test and are shown as mean (SD: standard deviation) when normally distributed, and nonparametric data are presented as median (Q1–Q3). The frequencies of categorical variables were compared using Fisher’s exact test, whereas continuous variables between groups were compared using Student’s *t*-test when normally distributed or the Mann–Whitney U test otherwise. Statistical significance was set at *p* < 0.05.

The learning curve for each group was analyzed using the cumulative sum (CUSUM) method for the surgery time. The CUSUM statistic for the first case in each group was calculated by comparing the surgery time with the median surgery time for all cases in the group.

Although there is no gold standard for the assessment of the learning curve, we used the time for the CUSUM curve and adjusted this curve, including, in the first stage, the variables tumor size, lymph node involvement, centrality, respiratory functional capacity, and use of anticoagulants. In the RATS group, by backward stepwise selection, the following variables remained in the adjustment model: tumor size, lymph node involvement, centrality, functional respiratory capacity, and use of anticoagulants. Similarly, in the VATS group, the adjustment model included tumor size, lymph node involvement, and centrality, as determined by backward stepwise selection.

We also created a variable called “surgical failure”, defined as the occurrence of at least one of the following factors: conversion, complications, readmission within 30 days, and mortality.

A univariate analysis was conducted to examine potential factors associated with surgical failure. The following variables were taken into consideration: age, gender, smoking history, presence of comorbidities, such as chronic obstructive pulmonary disease (COPD), hypertension (HT), diabetes mellitus (DM), chronic renal insufficiency (CRI), and cardiovascular disease (CV). Additionally, factors such as the use of anticoagulants, forced expiratory volume in one second (FEV1%), forced vital capacity (FVC%), diffusing capacity for carbon monoxide (DLCO), tumor size, induction, centrality, TNM stage, and lymph node involvement were included in the analysis.

Surgical time, conversions, complications, days of thoracic drainage, the length of stay, readmissions, and mortality at 90 days were compared between the two groups.

## 3. Results

### 3.1. Patient Demographics and Characteristics

Sixty-five lung resections were included in the analysis, 30 in the RATS group and 35 in the VATS group. The patient demographics and characteristics are summarized in Table 1. There were no statistically significant differences except for FEV1%, which was slightly higher in the RATS group (*p* = 0.035).

### 3.2. Learning Curve

**Figure 2:**
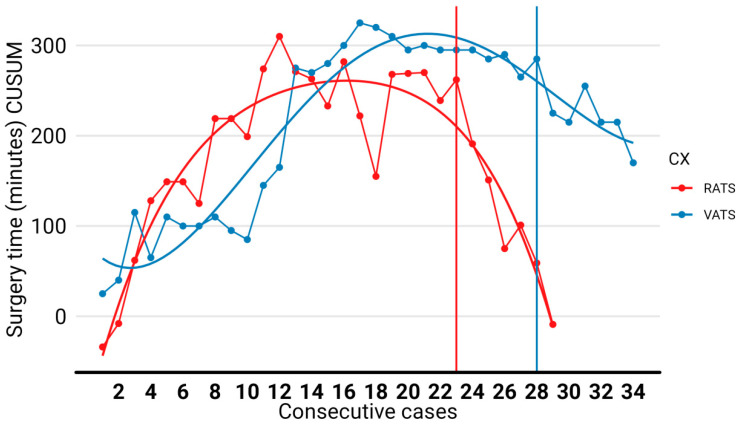
CUSUM curve, for the surgery time of the lobectomies. The RATS learning curve was completed at 23 procedures while VATS required 28 interventions. Abbreviations: RATS: robot-assisted thoracic surgery; VATS: video-assisted thoracic surgery.

**Figure 3:**
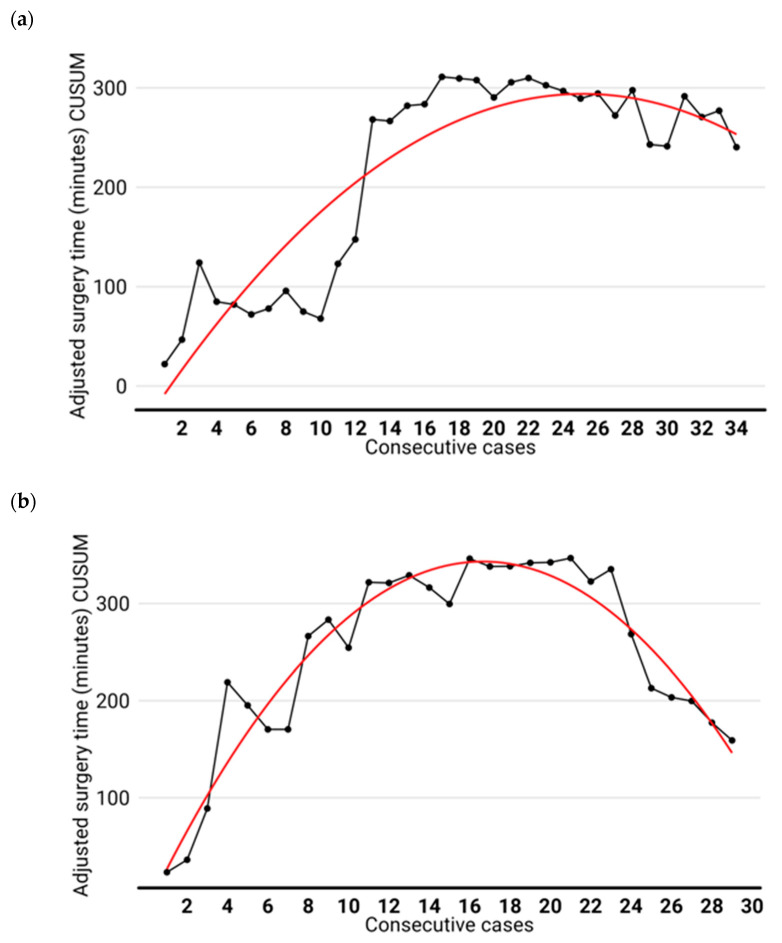
Adjusted CUSUM curve, for the surgery time of the lobectomies. The RATS learning curve (**a**) was completed at 23 procedures while VATS (**b**) required 31 interventions. Abbreviations: RATS: robot-assisted thoracic surgery; VATS: video-assisted thoracic surgery.

The median operative time was similar in both groups: 204 min (165–230) for the RATS group and 190 min (180–210) for the VATS group (*p* = 0.772). Regarding the learning curve analysis, in the RATS group, proficiency was obtained with 23 cases while in the VATS group, 28 procedures were required (Figure 2). When the curve was adjusted using the variables tumor size, lymph node involvement, centrality, respiratory functional capacity, and the use of anticoagulants, the differences increased. While the RATS group remained unaltered, the VATS group completed the curve at procedure number 31 (Figure 3).

### 3.3. Surgical Outcomes

**Figure 4:**
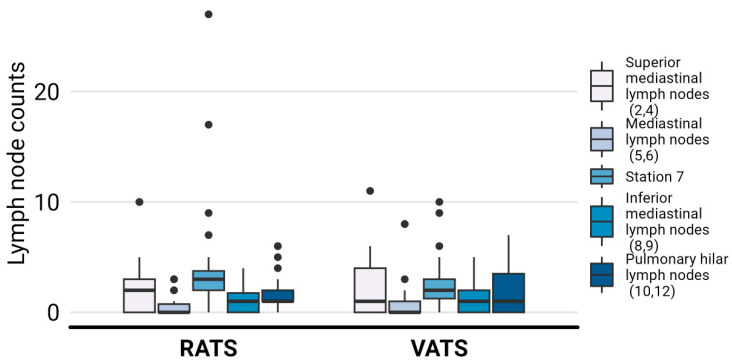
Lymph node counts for stations. Abbreviations: RATS: robot-assisted thoracic surgery; VATS: video-assisted thoracic surgery.

**Figure 5:**
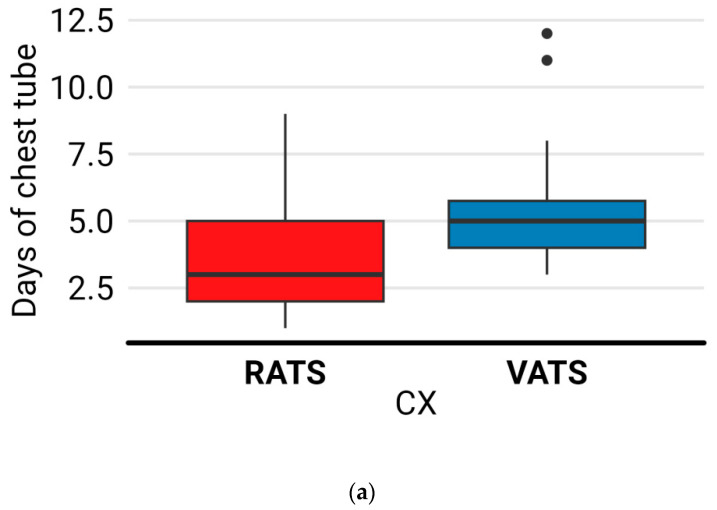

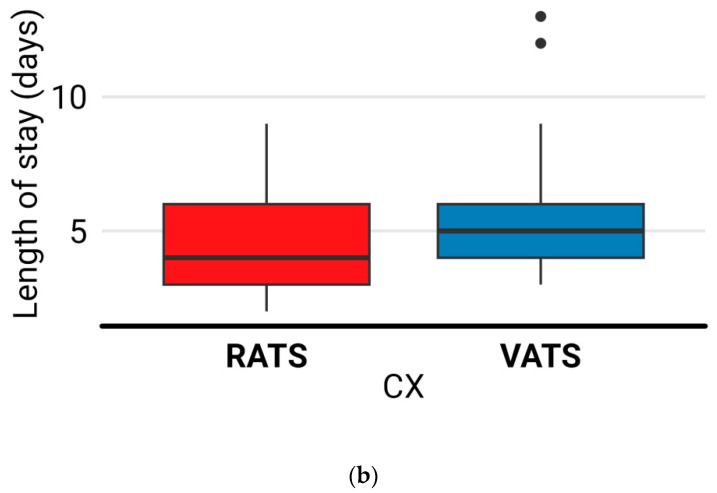
Comparison between days of chest tube (**a**) and length of stay (**b**) between groups. Abbreviations: RATS: robotic-assisted thoracic surgery; VATS: video-assisted thoracic surgery.

Conversion to thoracotomy was necessary for one patient in each group. In the RATS group, it was due to oncologic causes, and in the VATS group, it was required for technical reasons resulting from firm adhesions. Complications were registered in four patients (13.3%) in the RATS group: three of grade I (persistent air leaks) and one of grade III (bleeding requiring surgery) according to the Clavien–Dindo classification. The VATS group presented complications in eight patients (22.8%): five of grade I (five air leaks) and three of grade II (one case of anaphylactic shock, one of chylothorax, and one of bradycardia with heart block) (*p* = 0.358). Lymph node (LN) dissection: no differences in the number of LNs [9 (IQR, 7–12.8) vs. 9 (7–12.8), *p* = 0.738] and the harvested LN stations [five (IQR, four–five) vs. four (four–five), *p* = 0.842] was reported [14] (Figure 4). Among the 65 cases, five patients were upstaged from cN0 to pN1 (two in the RATS group and three in the VATS group), and only one patient in the RATS group was upstaged from cN0 to pN2. Among the patients in the RATS group, four individuals (13.3%) required readmission due to various reasons such as poor pain management, upper gastrointestinal bleeding, fever, and wound infection. On the other hand, in the VATS group, eight patients (22.8%) experienced readmission due to fever (two cases) as well as cardiovascular events, septic shock, pain, respiratory infection, and hemoptysis. We found that the RATS group had significantly shorter durations of chest drainage (median of 3 days (2–5)) compared to the VATS group (median of 5 days (4–5.8)), with a *p*-value of 0.005 (Figure 5a). Moreover, the RATS group also exhibited shorter hospital stays (median of 4 days, (3–6)) compared to the VATS group (median of 5 days (4–6)), with a *p*-value of 0.023 (Figure 5b).

No 90-day mortality was observed in either group. There was no significant association between the type of surgery and the occurrence of surgical failure (*p* = 0.29), with 8 (26.6%) in the RATS group and 14 (40%) in the VATS group. Surgical outcomes are summarized in Table 2.

## 4. Discussion

In our study, no differences were detected in terms of intraoperative time, intraoperative complications, conversions, postoperative complications, readmissions, and mortality when comparing VATS and RATS learning curves periods. In addition, in the RATS group, chest tube days and the total length of stay were significantly shorter.

The surgical time in oncological lung resections is mainly composed of two procedures: anatomical lung resection and lymphadenectomy. Surgical time is similar in both procedures. In addition, no differences in the number of lymph nodes removed and number of stations were detected when VATS and RATS approaches were compared. Furthermore, a similar upstaging of 10% in the RATS group and 8.57% in the VATS group in our series agreed with previously published data, and it is considered a technical quality indicator [15,16].

The first reported results for robotic anatomical resections pointed out long operative times [5,17,18], increased or no difference in the types or frequency of complications [6], air leak [19], and a long hospital length of stay [20] when compared to those of VATS. However, these studies typically compared an established VATS program to a recently developed robotic program. In our series of procedures conducted during the learning curves of both surgical approaches, these differences disappeared. In later years, as the novel robotic approach continued to evolve, the Portal study included and matched cases of more experienced surgeons performing both types of procedures [21]. The analysis favored RATS in terms of shorter durations of chest tube placement and the hospital length of stay. These findings reinforced our results and suggested that the superior outcomes observed during the learning curve will likely be sustained over time.

The concept of a learning curve is defined as the training period needed to achieve proficiency at a given technique. In their 2013 consensus statement, the international VATS lobectomy consensus group indicated that 50 operations should be performed in order to attain adequate technical competence, with at least 20 procedures performed annually to maintain operative skills [2]. In the study conducted by Mazzella et al., it was concluded that following the initial 30 lobectomies, the oncologic quality of the procedure exhibited improvement and stabilization. Subsequently, the surgeon demonstrated reduced selectivity and was willing to proceed with more complex cases, such as those involving incomplete fissures and pleural adhesions. Enhanced efficiency was achieved after 90 lobectomies, as evidenced by shorter operative times and a decreased conversion rate [22].

Focusing on the RATS lobectomy learning curve, work by B.N. Arnold et al. demonstrated a learning curve based on a surgical time of 22 operations, achieving mastery of the technique after 63 operations [23]. Song et al. conducted a study on a series of 208 patients using the CUSUM method based on docking duration, console time, and the total length of the intervention. The learning curve was established at 20, 34, and 32 cases, respectively [24]. These findings closely resembled our results, indicating that a comparable number of interventions is required for the successful implementation and consolidation of robotic programs, but in our series, the docking time remained almost constant, so it did not have an impact on the learning curve. Other groups required even fewer surgeries. Toker et al. analyzed the results of 102 robotic anatomical resections, including lobectomies and segmentectomies, and established a learning curve based on the operation duration for 14 cases [25]. Meyer et al. reached similar conclusions. In a series of 185 patients based on surgical time, mortality, and surgeon comfort, the learning curve was set at 15, 20, and 19 cases, respectively [26]. In a recent study conducted by the Division of Thoracic Surgery at the European Institute of Oncology in Milan, Italy, the learning curve of robotic lobectomies for lung cancer was assessed, considering the intraoperative cardiovascular and respiratory outcomes of the surgeon, which were recorded during surgical interventions to evaluate cardiovascular stress. The analysis indicated that confidence, competence, dexterity, and security were attained after approximately 20–30 procedures, without compromising efficiency and oncological radicality [27].

In the literature, we see there is no gold standard for measuring the learning curve, and we endorse the view that surgical time, though significant in its own regard, does not present a complete picture. Therefore, we adjusted our model for variables that could impact surgical time, such as tumor size, lymph node involvement, and centrality. The results showed a slight lengthening of the VATS curve while the RATS curve showed no changes. We also used the variable “surgical failure” described by Zhang et al. [28], which takes into account conversions, complications, 30-day readmissions, and mortality, but we found no significant association between the type of surgery and the occurrence of surgical failure. All in all, our findings were in range with the results of previous studies.

In our opinion, it is essential to monitor learning curve results closely when implementing a minimally invasive thoracic surgery program using the adjusted CUSUM curves. This quality control method allowed us to quickly spot variations in the curve and alerted us to the need of reviewing our procedures. Finding which variable is affecting the results enabled us to adjust and consequently provide better care for our patients [29].

One of the direct benefits our research highlights is the reduction in hospital days, which decreases the overall cost of robotic surgery. The most experienced centers are showing a progressive decrease in the number of days of stay [30], so we believe that this is a parameter to be considered in the economic calculations when investing in the implementation of this technology. Along these lines, a recent study by Harrison et al. concluded that completion of the learning curve was linked to a significant reduction in operating room costs associated with RATS lung resection and was comparable to the cost of VATS [31]. All these findings suggest that the economic gap between both procedures has begun to close as robotic surgery programs become more established.

Our study on robotic thoracic surgery acknowledged certain limitations. The study drew data from a single surgeon and the subgroup sizes for the different surgical techniques were relatively small. Moreover, it is reasonable to think that previous experience in video thoracoscopy and the technological advantages inherent in the robot may have played a role in the posterior robotic learning curve. In this sense, the surgeon who started the robotic procedures was more skilled than when he started VATS. These issues must be taken into account in order to design optimal training programs that reduce the learning curve to a minimum.

## 5. Conclusions

Regarding the learning curve analysis, in the RATS group, the curve was completed earlier than in the VATS group, at 23 and 28 procedures, respectively. When the curve was adjusted, while the RATS group remained unaltered, the VATS group completed the curve at procedure number 31. Moreover, during the training period, RATS exhibited a number of better short-term outcomes, including fewer days of chest drainage and reduced hospital stays in equal oncological quality resections. However, further investigations are required at a multicenter level to validate these results. Additionally, there is a need to elucidate the influence of prior VATS experience on the learning curve for RATS.

## Figures and Tables

**Table 1:** Patient demographics and characteristics.

	RATS (*n* = 30)	VATS (*n* = 35)	*p* Value
Age * years	65.69 (9.85)	70.41 (9.58)	0.059
Gender; *n* (%)			0.134
Female	16 (53.3)	12 (34.3)	
Male	14 (46.7)	23 (65.7)	
Smoker; *n* (%)			0.917
Active	11 (37.9)	11 (32.4)	
Former	6 (20.7)	8 (23.5)	
Never	11 (34.5)	12 (32.4)	
Unknown	2 (6.9)	4 (11.8)	
Comorbidity_yes; *n* (%)	18 (60)	22 (62.8)	1
COPD; *n* (%)	5 (16.6)	7 (20)	1
HT; *n* (%)	16 (53.3)	22 (62.8)	0.435
DM; *n* (%	5 (16.6)	10 (28.5)	0.375
CRI; *n* (%)	4 (13.3)	3 (8.5)	0.694
CV; *n* (%)	9 (30)	12 (34.2)	0.793
Anticoagulant; *n* (%)	6 (20)	6 (17.1)	1
FEV1_L *	2.29 (0.69)	2.35 (0.74)	0.756
FEV1_% *	88 (17.87)	78.59 (16.78)	0.035
FVC_L ^†^	3.20 (2.70, 4.09)	3.09 (2.66, 3.99)	0.847
FVC_% *	93.34 (15.81)	86.88 (15.68)	0.109
DLCO% ^†^	83 (77, 90)	77 (68, 91)	0.408
Tumor location; *n* (%)			0.694
Right upper lobectomy	11 (36.6%)	13 (37.1%)	
Right middle lobectomy	2 (6.6%)	1 (2.85%)	
Right lower lobectomy	5 (16.6%)	10 (28.5%)	
Left upper lobectomy	7 (23.3%)	8 (22.8%)	
Left lower lobectomy	5 (16.6%)	3 (8.57%)	
Tumor size; cm (SD)	1.70 (1.30, 2.50)	2.30 (1.33, 3.00)	0.307
Preoperative induction treatment; *n*	0 (0%)	1 (2.85%)	1
Centrality; *n*	2 (6.6%)	2 (5.7%)	1
Number of LNs; *n* (SD)	9 (7, 12.8)	9 (7, 12.8)	0.738
Sampled LNs stations; *n* (SD)	5 (4, 5)	4 (4, 5)	0.174
Superior mediastinal LN; *n* (SD)	2 (0, 3)	1 (0, 4)	0.690
Station 7; *n* (SD)	3 (2, 3.8)	2 (1.2, 3)	0.189
Mediastinal LNs; *n* (SD)	0 (0, 0.8)	0 (0, 1)	0.273
Inferior mediastinal LNs; *n* (SD)	1 (0, 1.8)	1 (0, 2)	0.814
Pulmonary hilar LNs; *n* (SD)	1 (1, 2)	1 (0, 3.5)	0.773
pStage TNM 8th			1
I; *n*	20 (76.9%)	25 (73.5%)	
II; *n*	5 (19.2%)	7 (20.6%)	
III; *n*	1 (3.8%)	2 (5.9%)	

* Data are presented as mean (SD), ^†^ Data are presented as median (Q1, Q3). Comparison of descriptive characteristics between groups. Abbreviations: RATS: robotic-assisted thoracic surgery; VATS: video-assisted thoracic surgery; COPD: chronic obstructive pulmonary disease; HT: hypertension; DM: diabetes mellitus; CRI: chronic renal insufficiency; CV: cardiovascular disease; FEV1: forced expiratory volume in one second; FVC: forced vital capacity; DLCO: diffusing capacity for carbon monoxide; LNs: lymph nodes; SD: standard deviation.

**Table 2:** Surgical outcomes.

	RATS (*n* = 30)	VATS (*n* = 35)	*p* Value
Operative time; minutes (range)	204 (165–230)	190 (180–210)	0.772
Learning curve completion; *n*	23	28	
Adjusted learning curve completion; *n*	23	31	
Surgical failure; *n* (%)	8 (26.6%)	14 (40%)	0.292
Conversion; *n* (%), causes	1 (3.3%) Oncologic causes	1 (2.8%) Firm adhesions	
Complications; *n* (%)	4 (13.3%)	8 (22.8%)	0.358
Grade I; *n* (%)	3 grade I (persistent air leaks)	5 grade I (air leaks)	
Grade II; *n* (%)	0	3 grade II (Anaphylactic shock, chylothorax, bradycardia with heart block)	
Grade III; *n* (%)	1 grade III (bleeding requiring surgery)	0	
Grade IV; *n* (%)	0	0	
Readmission; *n* (%)	4 (13.3%)	8 (22.8%)	0.358
Readmission causes	Difficult pain control Upper Gastrointestinal Bleeding, Fever Wound Infection	Fever in two cases Cardiovascular event, septic shock, pain, respiratory infection hemoptysis	
Days of chest drainage; days (range)	3 (2–5)	5 (4–5.8)	0.005
Length of stay; days (range)	4 (3–6)	5 (4–6)	0.023
90-day mortality; *n*	0	0	1.000

Abbreviations: RATS: robotic-assisted thoracic surgery; VATS: video-assisted thoracic surgery.

## Data Availability

The pseudonymized data presented in this study are available on request from the corresponding author. The data are not publicly available due to legal restrictions in Spain.
